# Efficacy of radiation boost after breast-conserving surgery for breast cancer with focally positive, tumor-exposed margins

**DOI:** 10.1093/jrr/rraa005

**Published:** 2020-03-12

**Authors:** Ryoko Suzuki, Masahiro Yoshida, Masahiko Oguchi, Yasuo Yoshioka, Kenji Tokumasu, Tomo Osako, Shinji Ono, Takayuki Ueno, Yumi Miyagi

**Affiliations:** 1 Department of Radiation Oncology, The Cancer Institute Hospital of JFCR, Tokyo, Japan; 2 Department of Radiology, Toho University Ohashi Medical Center, Tokyo, Japan; 3 Department of Pathology, The Cancer Institute Hospital of JFCR, Tokyo, Japan; 4 Department of Breast Oncology Center, The Cancer Institute Hospital of JFCR, Tokyo, Japan

**Keywords:** breast-conserving surgery, whole-breast radiation therapy, tumor-exposed margin, tumor bed boost, ductal carcinoma *in situ*, invasive breast cancer

## Abstract

Many patients with positive margins following breast-conserving surgery (BCS) undergo re-excisions that aim to remove residual disease from the breast, which brings a tremendous emotional burden in addition to financial consequences. We sought to determine whether re-excisions could be safely avoided without compromising local control and survival by using whole-breast radiation therapy (WBRT) with a tumor bed boost in patients with early-stage breast cancer with focally positive, tumor-exposed margins after BCS. All patients with ductal carcinoma *in situ* (DCIS) and/or invasive breast cancer (IBC) who had pathologically tumor-exposed margins following BCS, without re-excision and treated with WBRT with tumor bed boost between March 2005 and December 2011, were included. The radiotherapy consisted of WBRT at a dose of 50 Gy in 25 fractions, followed by a tumor bed boost with an additional dose of 16 Gy in eight fractions. A total of 125 patients fulfilled the eligibility criteria; of the 125 patients, 1 had bilateral breast cancer, resulting in 126 cases. Invasive disease was found in 102 (81%) cases and purely ductal carcinoma *in situ* (DCIS) disease in 24 (19%) cases. The 10-year ipsilateral breast tumor recurrence (IBTR) -free survival, progression-free survival (PFS), and 10-year overall survival (OS) rates were 95%, 92.5% and 96%, respectively. Patients with early-stage breast cancer who receive BCS and have focally positive, tumor-exposed margins can avoid re-excision by undergoing WBRT followed by a sufficient dose of tumor bed boost, without negatively impacting local control and survival.

## INTRODUCTION

Breast-conserving therapy (BCT) comprises complete surgical excision of the primary tumor with a clear margin followed by whole-breast radiation therapy (WBRT). However, a considerable proportion of patients undergo re-excisions during attempts to remove residual disease from the breast. The proportion of patients who undergo re-excisions for close and/or positive margins following lumpectomy is 22.9–26%, while the proportion who receive mastectomy is 8.5%–11.9% [1, 2]. The need for re-excision carries many drawbacks for patients, including worse cosmetic, financial, and emotional consequences. Insufficient surgical margins might be an indicator for higher rates of recurrence, but re-excisions and redundant margins might result in unnecessary procedures at the cost of a tremendous additional burden for patients.

The European Organization for Research and Treatment of Cancer (EORTC) ‘boost versus no boost’ trial showed that ipsilateral breast tumor recurrence (IBTR) at 10 years could be reduced with a boost dose of 16 Gy following WBRT in all age groups, although tremendous gains could be achieved in younger age groups (≤40 years of age) [3]. Furthermore, a 20-year follow-up of this trial confirmed the initial findings [4]. In our institution, we have long adapted a strategy to omit re-excisions and proceed with WBRT followed by a tumor bed boost for those patients with early-stage breast cancer with focally positive, tumor-exposed margins (i.e. tumors on ink in two or less foci on the cut-end), which deviates from international treatment guidelines concerning ductal carcinoma *in situ* (DCIS) and invasive breast cancer (IBC) [5–7].

In one study that reviewed re-excised specimens following an initial lumpectomy, the incidence of residual disease was not significantly different between focally positive margins (≤ 4-mm length of tumor touching inked margins) compared with close margins (<2-mm width) [8]. We believe that an adequate boost dose to the tumor bed following WBRT may be sufficient to eradicate any residual tumor at the surgical margin if the amount of residual disease is limited; therefore, we implemented our treatment strategy for focally positive, tumor-exposed margins. However, there is a clear deficiency of knowledge regarding the feasibility of this approach, because only a limited number of retrospective studies are available [9].

The aim of this study was to assess the efficacy of WBRT followed by tumor bed boost for patients with early-stage breast cancer with focally positive, tumor-exposed margins after they have undergone breast-conserving surgery (BCS). Moreover, we sought to determine whether re-excisions could be safely avoided without compromising local control and even survival for these patients.

## MATERIALS AND METHODS

Following approval by the appropriate institutional review board, participants for this study were identified from the database of breast cancer patients who had undergone BCS and WBRT between March 2005 and December 2011 at a single institution. Patients were eligible if their postoperative pathological report included IBC and/or DCIS with 0-mm margins (i.e. ink on tumor) and were excluded in cases of >0-mm margins (i.e. no ink on tumor) and/or receipt of neo-adjuvant chemotherapy. At our institution, BCS is performed by oncological surgeons specializing in breast cancer, who aim to excise a tumor with a 1-cm macroscopic margin. The entire thickness of breast tissue, from subcutaneous fat down to the pectoral fascia, is removed in all patients.

Partial mastectomy materials were continuously sectioned from the nipple side to the periphery at 5-mm intervals [10]. All sections were histologically examined with hematoxylin-and-eosin staining by pathologists specializing in breast cancer. The pathology reports included microscopically measured distances from cancer nests to the surgical margins (i.e. lateral, superficial and deep margins).

Our general approach is to recommend re-excision in cases of extensively positive margins (i.e. tumor-exposed margin 0 to < 5 mm, with multiple foci, i.e. three or more foci involvement) or proceed with WBRT with lumpectomy boost in cases of focally positive margins (i.e. margin of tumor-exposed 0 to < 5 mm with focal foci such as two or less foci involvement). Therefore, only those patients with focally positive, tumor-exposed margins were included in this cohort. However, due to the retrospective nature of this study, those patients with extensively positive margins may have been included in this cohort. Although our general approach for these patients was as mentioned above, some patients refused re-excisions despite our recommendation.

All patients in this cohort received WBRT at a dose of 50 Gy in 25 fractions, followed by a tumor bed boost with an additional dose of 16 Gy in eight fractions. Adjuvant endocrine therapy was given for patients with hormone receptor–positive invasive cancers: tamoxifen for 5–10 years with or without 5 years ovarian function suppression based on the risk for premenopausal women; aromatase inhibitors for 5 –10 years depended on the risk for postmenopausal women.

Patients generally returned for follow-up every 3 months for the first 2 or 3 years, every 6 months from the third through the fifth years, and yearly thereafter, although variations did exist.

Baseline demographic, tumor and treatment characteristics were obtained from the electronic medical records of each patient. For the analyses of IBTR, progression-free survival (PFS) and overall survival (OS), time to event was counted from the date of surgery to the date of IBTR, to the date of first progression at any site, and to the date of death, respectively. Analyses were performed using IBM SPSS Statistics version 22.0.

## Results

A total of 125 patients fulfilled the eligibility criteria; of the 125, 1 patient had bilateral breast cancer, resulting in 126 cases. Within this cohort, 7 patients underwent a second surgery to re-excise remaining tumor due to extensively tumor-exposed margins, although again tumor remained as focally positive tumor-exposed margins. Baseline patient, tumor and treatment characteristics of the participants are listed in Table 1.

**Table 1:** Patient, tumor and treatment characteristics

Characteristics	All cases (*N* = 126) (%)
*Patients*	
Median age (IQR) (years)	52 (43–64)
History of contralateral breast cancer	
Yes	8 (6)^a^
No	118 (94)
Pre-operative MRI	
Yes	118 (94)
No	8 (6)
*Tumor*	
Purely DCIS disease	24 (19)
Invasive disease	102 (81)
Size of invasive disease (cm)	
No invasions	24 (19)
>0, ≤0.1	11 (9)
>0.1, ≤0.5	12 (10)
>0.5, ≤ 1	17 (13)
>1, ≤2	47 (37)
>2	13 (10)
N/A	2 (2)
Nuclear grade	
1	40 (32)
2	34 (27)
3	12 (10)
N/A	40 (32)
ER	
Positive	113 (90)
Negative	12 (10)
N/A	1 (1)
PgR	
Positive	89 (71)
Negative	36 (29)
N/A	1 (1)
HER2	
Positive	38 (30)
Negative	72 (57)
N/A	16 (13)
Lymphovascular invasion	
Yes	29 (23)
No	97 (77)
Fat tissue invasion	
Yes	80 (63)
No	46 (37)
No. of positive nodes	
0	103 (82)
1–3	20 (16)
4–9	3 (2)
Multifocality	
Unifocal	116 (92)
Multifocal	10 (8)
Feature of tumor-exposed margin	
*In situ* disease	107 (85)
Invasive disease	15 (12)
Both *in situ* and invasive disease	4 (3)
Site of tumor-exposed margin	
Lateral	79 (63)
Superficial	25 (20)
Deep	9 (7)
Lateral + Superficial/Deep	12 (10)
Superficial + Deep	1 (1)
No. of tissue specimen slices that contained tumor-exposed cut ends	
1	88 (70)
2	27 (21)
3	7 (6)
4–6	4 (3)
No. of tissue specimen slices that contained close margin (<5 mm width)^b^	
1	24 (19)
2	28 (22)
3	23 (18)
4	26 (21)
5–7	19 (15)
8–10	6 (5)
*Treatment*	
*Systemic therapy*	
Receipt of adjuvant endocrine therapy	91 (72)
Receipt of adjuvant chemotherapy	32 (25)
*Radiation therapy*	
Median time (days) between date of surgery to RT start date (IQR)	86 (72–170)

IQR = interquartile range, MRI = magnetic resonance imaging, DCIS = ductal carcinoma *in situ*, N/A = not applicable, ER = estrogen receptor, PgR = progesterone receptor, HER2 = human epidermal growth factor receptor 2, RT = radiation therapy.

^a^Included four patients with concurrent bilateral breast cancer.

^b^Included tumor-exposed cut ends.

The majority of patients (94%) underwent pre-operative magnetic resonance imaging (MRI) scans. Invasive disease (with or without contiguous intraductal components) was found in 102 (81%) cases and purely DCIS disease in 24 (19%) cases. For management of axillary lymph nodes, sentinel lymph node biopsies were performed in 96 (76%) cases, level 1 axillary lymph node dissection in 4 (3%) cases, level 2 axillary lymph node dissection in 19 (15%) cases, and the remaining 7 (6%) cases had no surgical assessment of the axilla. Many patients (82%) had node-negative disease, although lymphovascular invasion (including slight invasion) was seen in 29 patients (23%). Only 10 patients (8%) had multifocal tumors. A preponderance of tumor-exposed margins occurred with *in situ* disease only (85%). The remaining 15% of cases had invasive disease or both *in situ* and invasive disease. The lateral margin was the most frequent site of a tumor-exposed margin (63%). Most patients (91%) had focally positive tumor-exposed margins, with only one or two tissue-slice specimens that contained tumor-exposed cut-ends. Of 102 patients with invasive disease, only 19 patients (19%) had tumor-exposed margins with invasive features.

The median follow-up time for all patients was 114 months (interquartile range 96–122 months). During follow-up, six patients were diagnosed with IBTR: five of those patients had recurrence only at the tumor bed; the other had recurrence at the tumor bed and also had recurrence as diffuse breast and skin invasion in different area of the affected breast; furthermore, she had simultaneous distant metastasis to the contralateral axillary lymph node. Four patients showed distant metastasis of whom one patient had simultaneous IBTR, as mentioned above. The overall 10-year IBTR cumulative incidence was 5%: 0% for the purely DCIS cohort; 6% for the IBC cohort.

**Fig. 1.**
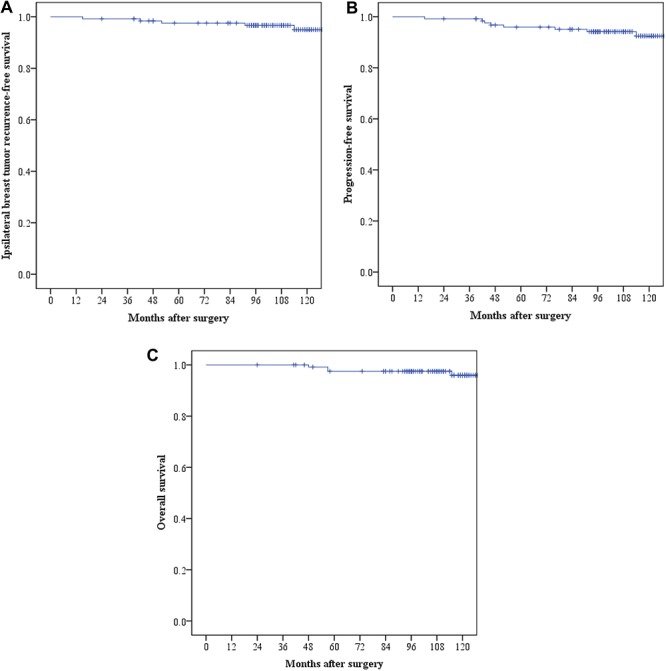
(**a**) Kaplan–Meier plot of ipsilateral breast tumor recurrence (IBTR); (**b**) progression-free survival (PFS); and (**c**) overall survival (OS).

Five deaths were observed: three patients died of breast cancer and two patients died of other causes. The 10-year IBTR-free survival, PFS and OS rates were 95%, 92.5% and 96%, respectively. Survival curves are shown in Fig. 1.

## Discussion

In this study, we showed that patients with early-stage breast cancer with focally positive tumor-exposed margins who underwent BCS safely avoided re-excision by receiving WBRT followed by a tumor bed boost of a dose of 16 Gy; this avoidance of re-excision could be achieved without disturbing local control or patient survival.

Associations between positive margins and IBTR have been reported in many studies of both DCIS [11, 12] and IBC [13]. In the Early Breast Cancer Trialists’ Collaborative Group’s meta-analysis of randomized DCIS trials, patients with positive margins were shown to bear twice as high a risk of IBTR compared with patients with negative margins, even if they received WBRT (10-year ipsilateral breast event, 24.2% vs 12.0%), and approximately half of those were invasive recurrences [11]. In a subset analysis of the National Surgical Adjuvant Breast and Bowel Project (NSABP) B-17 trial, central pathology observation found nine pathologic features (including margin status) in 623 of 814 participants. The annual hazard rate of IBTR after lumpectomy alone was 7.2% for those with positive margins, compared with 4.7% for those with negative margins; these rates were curtailed by WBRT to 2.5% and 1.9%, respectively [12]. In a meta-analysis of 33 studies of patients treated with BCT for IBC, local recurrences were reported in 1506 of 28 162 individuals (5.3%). With regard to margin status, the odds ratio (OR) of IBTR for positive versus negative margins was 2.44 [95% confidence interval (CI) 1.97–3.03] [13]. In a previous study with the cohort of Japanese patients that included both DCIS and IBC, the 10-year cumulative rates of IBTR were 8.5% in patients who received WBRT, while those patients with positive surgical margins had approximately a 3-fold increased risk of IBTR compared with negative margins [14]. Another study with a Japanese cohort had similar findings [15]. In our study, the overall 10-year cumulative incidence for IBTR was 5%: 0% for the purely DCIS cohort and 6% for the IBC cohort, which is comparable with results of the previous studies that included patients with positive and negative margins.

Although BCT is extensively used to treat early-stage breast cancer, there has long been controversy and disagreement as to what constitutes an optimal surgical margin [16, 17]. Accordingly, approximately one-third of women who receive BCT for DCIS or IBC also have to endure re-excision [1, 2]. One observational study showed that among 1459 women for whom BCS was undertaken, additional surgery was required in 37.9% of patients, including 358 who underwent re-excision (26%) and 167 who had mastectomies (11.9%) [2]. In patients with DCIS, a negative surgical margin should be obtained regardless of whether they received radiotherapy. A previous meta-analysis, which included studies of DCIS published between 1970 and 2010, recommended wider free margins of up to 10 mm, within cosmetic constraints, whenever possible [18]. However, larger margins are certainly detrimental in terms of patients’ cosmetic outcomes. Our hypothesis is that an adequate tumor bed boost following WBRT nullifies the negative prognostic impact of positive margins. Furthermore, we showed that we could avoid re-excision without compromising local control and survival in those patients with focally positive tumor-exposed margins. If our approach for positive margins were to be implemented, the number of patients needing re-excisions could be reduced.

Substantial surgeon and institutional variations were observed for re-excisions following lumpectomy [1]. There is inconsistency in patterns of practice and physician interpretation with regard to what constitutes an adequate surgical margin [16, 17]. In a study by Blair *et al*., when surgeons were asked what they considered to be acceptable margins for DCIS and IBC, a large range of answers was obtained: 15% of surgeons considered the margin of the tumor acceptable if any negative margin was present, while 28%, 50%, 12% and 3% considered negative margins of 1, 2, 5 and 10 mm, respectively, to be necessary [16]. Azu *et al.* also conducted a survey among surgeons who had conducted BCS between 2005 and 2007, in which surgeons were given a scenario of T1 (0.8 cm) invasive cancer; 11% of surgeons approved margins of tumor not touching ink, 42% approved margins of 1–2 mm, 28% approved margins of ≥5 mm, and 19% approved margins of >1 cm as precluding the need for re-excision before radiotherapy [17]. The deficiency of a prevailing standard of care has resulted in making the margin issue tremendously controversial.

In 2016, the Society of Surgical Oncology (SSO), the American Society for Radiation Oncology (ASTRO) and the American Society of Clinical Oncology (ASCO) published guidelines for a lumpectomy margin in patients with DCIS undergoing BCT and concluded that the use of a 2-mm margin should be the standard for adequate margins in DCIS treated with BCS and WBRT [5]. Furthermore, SSO and ASTRO have also provided consensus guidelines on margins in patients with Stage I and II breast cancer treated with BCS followed by WBRT and concurred that no ink on the tumor should be used as the standard for an adequate margin [6, 7]. As part of their process for developing the guidelines, a multidisciplinary panel of SSO and ASTRO specialists conducted a systematic review of 33 published studies to determine the impact of margin width on IBTR in patients with IBC. They confirmed that positive margins were associated with a 2-fold increase in the risk of IBTR compared with negative margins. Moreover, by utilizing the description of no ink on tumor as a negative margin, there was no evidence that wider negative margins decreased the risk of IBTR.

In one series from the Netherlands that reviewed re-excised specimens following an initial lumpectomy, the incidence of residual disease was not significantly different after focally positive margins (classified as a ≤4-mm length of tumor touching inked margins) compared with close margins (classified as <2-mm width) [8]. The national breast cancer guidelines of the Netherlands advocate a treatment approach comparable with that of our institution as regards indications for re-excision according to tumor margins [19]. They advise re-excision only in cases of extensively positive margins (tumor touching the inked margin of more than 4-mm length or more than focal positive foci).

We have demonstrated that patients with early-stage breast cancer with focally positive tumor-exposed margins following BCS could avoid re-excision without any detrimental effects on local control or their survival. We showed that an adequate boost dose to the tumor bed following WBRT was sufficient to eradicate residual tumor at the surgical margins if the amount of residual disease is limited.

The median follow-up time in this study was 114 months. The Early Breast Cancer Trialists’ Collaborative Group stated that about three-quarters of the local recurrence risk following BCS occurred during the first 5 years, and that differences in 5-year local recurrence risk were comparable with 15-year breast cancer mortality [20]. Therefore, the results of this analysis are likely to be maintained over time.

Aside from the inherent limitations of any retrospective study, limitations in our study include heterogeneities in the site and number of tumor-exposed surgical margins. The general practice at our institution is to offer re-excision for patients with diffusely involved positive margins, such as three or more foci contained in tumor-exposed cut-ends. However, to offer WBRT with a tumor bed boost of 16 Gy without re-excision for patients with focally involved positive margins, such as two or less foci that contained tumor-exposed cut-ends, is a practice that is subject to substantial variations. Additionally, the features of tumor-exposed margins in this study were heterogeneous: purely DCIS, DCIS and IBC, or IBC only. Despite these limitations, we have analyzed patients with tumor-exposed positive margins only, using a relatively large dataset. Furthermore, our radiotherapy approach with these patients was uniform: WBRT with a tumor bed boost of 16 Gy with standard fractionation. Our treatment strategy should be prospectively validated in a randomized clinical trial, preferably conducted among multiple institutions, before this strategy is widely applied in clinical practice.

## Conclusions

Patients with early-stage breast cancer with focally positive, tumor-exposed margins following BCS can safely avoid re-excision by receiving WBRT followed by a tumor bed boost with a dose of 16 Gy without interfering with local control or their survival. This treatment strategy saves patients from the negative implications of re-excisions, including cosmetic, financial and emotional impacts.

## Acknowledgements

This work was presented at the 32nd Annual Meeting of the Japanese Society for Radiation Oncology (2019).

## Conflict of Interest

The authors declare that there are no conflicts of interest.
